# Molecular Interactions between Tarantula Toxins and Low-Voltage-Activated Calcium Channels

**DOI:** 10.1038/srep23894

**Published:** 2016-04-05

**Authors:** Autoosa Salari, Benjamin S. Vega, Lorin S. Milescu, Mirela Milescu

**Affiliations:** 1 University of Missouri, Division of Biological Sciences, Columbia, 65211, USA

## Abstract

Few gating-modifier toxins have been reported to target low-voltage-activated (LVA) calcium channels, and the structural basis of toxin sensitivity remains incompletely understood. Studies of voltage-gated potassium (Kv) channels have identified the S3b–S4 “paddle motif,” which moves at the protein-lipid interface to drive channel opening, as the target for these amphipathic neurotoxins. Voltage-gated calcium (Cav) channels contain four homologous voltage sensor domains, suggesting multiple toxin binding sites. We show here that the S3–S4 segments within Cav3.1 can be transplanted into Kv2.1 to examine their individual contributions to voltage sensing and pharmacology. With these results, we now have a more complete picture of the conserved nature of the paddle motif in all three major voltage-gated ion channel types (Kv, Nav, and Cav). When screened with tarantula toxins, the four paddle sequences display distinct toxin binding properties, demonstrating that gating-modifier toxins can bind to Cav channels in a domain specific fashion. Domain III was the most commonly and strongly targeted, and mutagenesis revealed an acidic residue that is important for toxin binding. We also measured the lipid partitioning strength of all toxins tested and observed a positive correlation with their inhibition of Cav3.1, suggesting a key role for membrane partitioning.

Voltage-gated calcium (Cav) channels are membrane proteins that facilitate communication via electrical and chemical signaling in a wide variety of cells and organisms. Importantly, Ca^2+^ is not only the permeant ion for Cav channels but is also a second messenger in cellular processes. Thus, by coupling an electrogenic role with a regulatory function, Cav channels orchestrate many physiological processes, such as muscle contraction, hormone secretion, neurotransmitter release, and regulation of gene expression^1^. Topologically, Cav channels are made of four covalently linked homologous domains (DI–DIV), each containing six transmembrane helices (S1–S6) that form the pore (S5–S6) and the four voltage-sensing domains (S1–S4). Although the four channel domains (DI–DIV) have similar sequences, there is significant variability within each channel and across channel types^2^. This variability may render the four voltage sensors functionally and pharmacologically distinct. For example, a recent study has characterized the unique voltage- and time-dependent properties of each voltage sensing domain in Cav1.2 channels, using optical and electrical recordings^3^. The evidence clarifies the role played by each domain in channel opening: domains II and III are the key players and they must activate for the channel to open, domain I has a minor contribution, whereas domain IV plays no role, as it activates on voltage and time scales incompatible with the overall channel opening. For low-voltage-activated (LVA) Cav3.1 channels, this heterogeneity within the voltage sensors has yet to be explored.

In the related homotetrameric Kv channels, a structural correlate (S3b–S4) for voltage sensing^4^,^5^,^6^ and pharmacology^7^,^8^,^9^,^10^,^11^,^12^ has been identified. This S3b–S4 segment (“paddle motif”) moves at the protein-lipid interface in response to changes in voltage and causes the channel to open^4^,^5^,^13^,^14^,^15^. Given its central role in voltage sensing, it is not surprising that the paddle is targeted by amphipathic gating-modifier neurotoxins that modulate the voltage-dependent activation of the channel^7^,^8^,^9^,^12^. All gating-modifier toxins known to date share an inhibitory cysteine knot (ICK) motif that stabilizes the overall structure and enables the formation of the binding site^16^,^17^. The binding site contains a large hydrophobic surface surrounded by charged amino acids^18^,^19^,^20^,^21^, which together are involved in toxin binding to the channel through the lipid bilayer^20^,^22^,^23^,^24^,^25^. For Kv channels, this tri-partite interaction between channel, toxin, and lipids has been well studied^23^,^26^,^27^ and utilized to shed light on channel function. However, much less is known about Cav channels, particularly LVA, for which the pharmacological repertoire is limited.

Kurtoxin and ProTx-II are among the few known LVA calcium channel gating-modifier toxins^17^,^28^,^29^,^30^. However, the molecular interactions between the toxins, target Cav3 channels, and lipid membranes are still unknown. In this study, we focus on ProTx-II, a toxin isolated from the *Thrixopelma pruriens* tarantula, which targets both Cav3.1 and Nav channels^31^. Like other gating-modifier toxins, ProTx-II slows activation kinetics, accelerates deactivation, decreases the macroscopic current, and shifts the activation curve (I–V) to more positive potentials in both channel types, with no significant effect on steady-state availability or recovery from inactivation^29^,^32^. The shift in the activation curve is dependent on both the toxin concentration and the divalent cation concentration in the extracellular recording solution. Thus, it has been shown that ProTx-II decreases current amplitude to a similar extent in both 2 mM Ca^2+^ and 5 mM Ba^2+^ solutions, but the shift in the I–V curve is three times smaller in the presence of Ba^2+^ vs. Ca^2+^, which was explained by a surface-charge screening mechanism of the toxin^29^. The decrease observed in macroscopic conductance is not caused by changes in single-channel conductance or by changes in the total gating charge. However, the voltage-dependent movement of gating charges (Q–V) is shifted towards more depolarizing voltages, suggesting interactions between the voltage sensors and the toxin. Overall, it has been proposed that the toxin interacts with multiple voltage sensors to modify several transitions along the activation pathway^29^. However, the voltage sensor structures involved in binding have yet to be identified. ProTx-II has also been shown to interact with lipids^33^, but the strength of lipid partitioning has not been tested.

Using chimaeric constructs of Cav voltage sensors embedded within a Kv2.1 host channel, we identified the paddle motif of each Cav3.1 domain and examined the domain-specific interactions with ProTx-II. In addition, we discovered that three other tarantula toxins, previously reported to inhibit Nav and Kv channels, are also gating-modifiers of Cav3.1 channels. Lastly, we probed the lipid partitioning strength of all four toxins by measuring intrinsic tryptophan fluorescence. Our results demonstrate that sequence differences across the four voltage-sensors cause functional heterogeneity in the voltage-sensing and pharmacological properties of LVA channels. In addition, only a few differences in the amino acid sequence of the toxins studied here can cause differences in channel inhibition and lipid partitioning. These two aspects, channel inhibition and lipid partitioning, seem to be positively correlated.

## Results

### Cav3.1 paddle motifs

It has been proposed that Cav channels, like their Kv and Nav relatives, also have functional paddle motif substructures within each voltage sensor^34^,^35^. However, it has not been confirmed yet whether they operate by the same structure/function principles as in Kv channels. To define structural and functional motifs in Cav3 voltage sensors, we transferred putative paddle-forming sequences from Cav3.1 into Kv2.1 channels, separately for each of the four domains. This approach has been used previously for potassium and sodium channels^11^,^36^. The rationale is that it is easier to study an unknown functional unit when embedded into the known structure of the Kv channel^37^. Furthermore, since the Kv host channel is homomeric, each chimaera is also a homomer and thus the four Cav S3–S4 segments can be analyzed individually.

**Figure 1:**
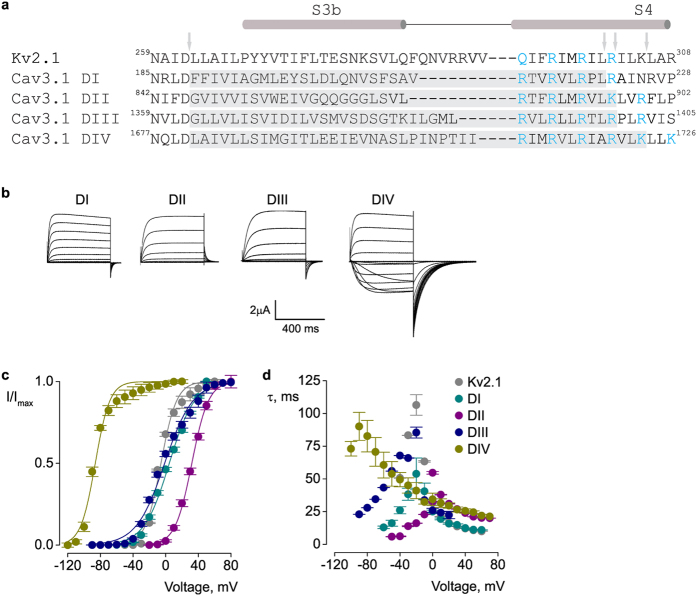
Paddle motifs in Cav3.1 voltage-sensor domains. (**a**) Sequence alignment of the paddle region of Kv2.1 and S3–S4 regions of the four Cav3.1 domains (DI–DIV)^2^. The conserved charged residues involved in voltage-sensing are shown in blue. Grey regions and arrows indicate the sequences that were swapped between the two channels to create paddle chimaeras. The numbers correspond to the amino acid residues within the parent channel. (**b**) Potassium currents for Cav3.1/Kv2.1 chimaeras. (**c**) Voltage-activation data and Boltzmann fits for Kv2.1 and Cav3.1/Kv2.1 paddle chimaeras. Conductance was determined from normalized tail currents. V_1/2_ values are (mV): 4.4 ± 0.9 (DI), 25.5 ± 0.7 (DII), **−**1.3 ± 1 (DIII), −87.1 ± 1 (DIV), and −5.8 ± 1 (Kv2.1). (**d**) Time constants (τ) of activation and deactivation determined from single exponential fits at each voltage. Data shown in (**b–d**) were obtained with the following voltage-step protocol: holding voltage −100 mV (Kv2.1 and DI-DIII) or −120 mV (DIV); test pulse duration 500 ms; tail voltage −60 mV (Kv2.1, DI and DIII), −10 mV (DII), or −120 mV (DIV). I/I_max_ (**c**) is the normalized tail current amplitude. In all panels, data points are mean ± SEM (n = 6).

Although the sequences of the S3−S4 regions vary substantially between Cav3.1 and Kv2.1 channels (Fig. 1a), we were able to define individual paddle motifs within each of the Cav3.1 domains and show that they can result in fully functional chimaeric channels that display robust, voltage-activated potassium currents (Fig. 1b). Interestingly, the four chimaeras display distinct gating properties (Fig. 1c). Relative to the host Kv2.1 channel, the half-activation voltage of domain II paddle chimaera is shifted to more positive values (ΔV_1/2_ = 31 mV), while the domain IV paddle chimaera exhibits a negative shift (ΔV_1/2_ = −81 mV). Domain I and III paddle chimaeras have half-activation voltages similar to Kv2.1 (Table 1). The four paddle chimaeras also exhibit distinct activation and deactivation kinetics (Fig. 1d).

**Figure 2:**
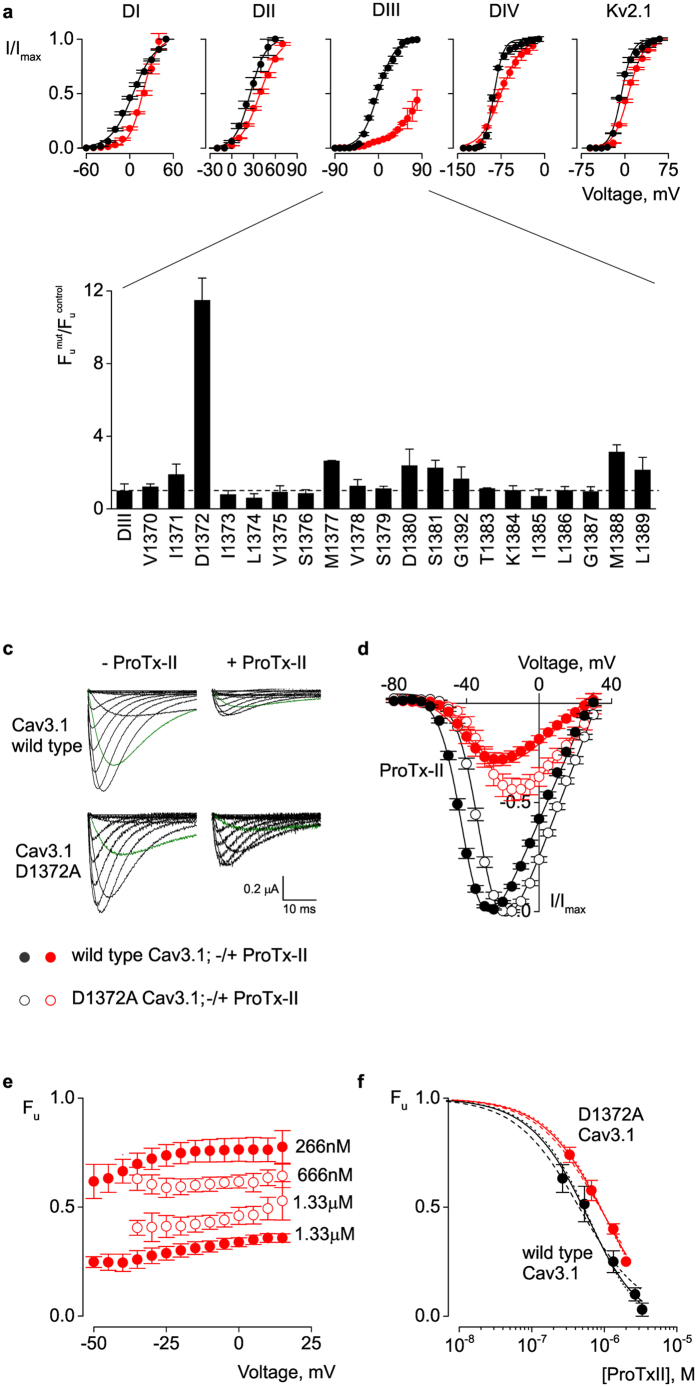
ProTx-II binding sites on Cav3.1 voltage-sensors. (**a**) Voltage-activation data and Boltzmann fits in the absence (black) and presence (red) of 1.33 μM ProTx-II for Kv2.1 and Cav3.1/Kv2.1 chimaeras. I/I_max_ is the normalized tail current amplitude. In the presence of toxin, the V_1/2_ values are (mV): 17.7 ± 1.8 (DI), 38.8 ± 0.7 (DII), **−**75.4 ± 1.4 (DIV), 6.3 ± 1 (Kv2.1). A meaningful fit could not be obtained for DIII. (**b**) Alanine scan of DIII chimaera. Fraction of uninhibited current in the presence of 1.33 μM ProTx-II for alanine mutants (F_u_^mut^) normalized to DIII construct (F_u_^control^). (**c**) Barium currents for wild type and D1372A Cav3.1 channels in the absence (left) and presence (right) of 1.33 μM ProTx-II. Current elicited at −40 mV is highlighted in green. (**d**) Voltage-activation relationships for wild type (solid circles) and D1372A Cav3.1 (open circles) in the absence (black) and presence (red) of 1.33 μM ProTx-II. Curves are Boltzmann fits with V_1/2_ values of −41 ± 0.4 mV and −31 ± 0.3 mV for wild type and D1372A channels, respectively, in the absence of toxin; and −34 ± 1 mV and −25 ± 1 mV, in the presence of toxin. (**e**) Fraction of uninhibited current of wild type (solid circles) and D1372 (open circles) Cav3.1 channels in the presence of two ProTx-II concentrations. (**f**) Dose-response data and fit curves for wild type (solid circles) and D1372A (open circles) channels. Data were fit with both *n* and K_d_ as free parameters (solid lines), or with K_d_ as free parameter and *n* constrained to 1 (dashed lines), 2, or 3 (dotted lines). With two free parameters, the best fit values are K_d_ = 1.30 ± 0.6 μM and n = 2.11 ± 0.8 for wild type, and K_d_ = 1.44 ± 0.3 μM and n = 1.45 ± 0.25 for D1372A. F_u_ was calculated at −40 mV for the wild type and −30 mV for the mutant channel. In all panels, data points are mean ± SEM (n = 6).

The observed differences in the voltage-sensing properties of the Cav3.1/Kv2.1 paddle chimaeras suggest that they could also have distinct pharmacological profiles. It has been proposed that ProTx-II inhibits Cav3.1 by modifying multiple gating transitions along the activation pathway of the channel, and thus it may be able to bind to more than one voltage sensor^29^. However, the exact target within each of the four voltage-sensors is not known. We screened each paddle chimaera for interactions with ProTx-II and measured the voltage-dependent activation (I–V) of the channel in the presence and absence of toxin. In the presence of toxin, domain III chimaera exhibits the largest decrease in the ionic current, followed by domain I chimaera. Because of ambiguity in the sigmoidal activation profile, it is difficult to interpret how much of the observed inhibition results from a positive shift in the activation V_1/2_ and how much results from a reduction in the maximum current (Fig. 2a). In agreement with previous reports^31^,^36^, we also found little inhibition of the background Kv2.1 channel by ProTx-II (Fig. 2a). Our results demonstrate, for the first time, that paddle motifs exist within the Cav3.1 channel and that they have distinct voltage-sensing and pharmacological properties.

### An acidic residue in domain III of Cav3.1 is important for ProTx-II activity

The large inhibitory effect exerted by ProTx-II on the domain III chimaera (Fig. 2a) suggests that the toxin binds with highest affinity to the domain III voltage sensor paddle of Cav3.1. To identify the residues within this domain that are critical for the interaction with ProTx-II, we performed alanine-scanning mutagenesis within the chimaeric construct. Previous studies have identified binding sites for gating-modifier toxins within the S3 regions of related voltage-gated channels^9^,^34^,^38^,^39^,^40^,^41^. For example, in the case of hanatoxin (HaTx), the hydrophobic (isoleucine and phenylalanine) and acidic (glutamate) residues in the S3b region of the Kv2.1 channel are the most important for toxin activity^9^. Thus, we created alanine mutants (S3 region V1370 to L1389) in the chimaeric constructs, and recorded potassium currents in the presence and absence of toxin. The gating properties of the mutant channels are shown in Table 1. When compared with the parent domain III chimaera, the alanine mutants show similar inhibition by toxin (Fig. 2b). The only one exception is D1372A, which is significantly less inhibited by toxin (8 fold increase in K_d_, Table 1). This mutant exhibits a measurable toxin-induced shift in the I–V curve (ΔV_1/2_ = +5 mV, data not shown). Interestingly, an acidic residue exists at the equivalent location in domains I (E198) and II (E855) (Fig. 1a). We changed these residues to alanine within the domain I and II chimaeric constructs and observed only a 3-fold decrease in inhibition by ProTx-II (Table 1), and toxin-induced shifts in the I–V curve of +10 mV and +3 mV, respectively (data not shown). It is also interesting that domain IV, which is arguably the least affected by ProTx-II, lacks an acidic residue at this position.

Next, we tested whether the D1372A mutation has a similar effect in the native Cav3.1 channel as well. For the wild type Cav3.1 channel, we find that 1.33 μM ProTx-II produces a +7 mV shift in the voltage-activation curve and a 75% decrease in the peak current (Fig. 2c,d, solid circles). To accurately determine the dose-dependence of channel inhibition, we measured the fraction of uninhibited current at negative voltages, across a range of ProTx-II concentrations (Fig. 2e,f). Assuming equal and independent binding sites, the best fit parameters (n = 2.11 ± 0.8, K_d_ = 1.3 ± 0.6 μM, Fig. 2f) suggest a 2:1 stoichiometry between toxin and channel. This is consistent with our chimaeric experiments and favors a scenario where ProTx-II binds with highest affinity to domains I and III of Cav3.1. To help the reader understand the statistical significance of the estimated n values, we provide additional fits where n was constrained to 1 or 3, and K_d_ was estimated (Fig. 2f).

In contrast, for the Cav3.1 D1372A domain III mutant, while 1.33 μM ProTx-II produces a similar shift in the voltage activation (+6 mV), the decrease in peak current is only 50% (Fig. 2c,d, open circles). Moreover, the dose-response curve is best described with n = 1.45 ± 0.25 and K_d_ = 1.4 ± 0.3 μM, which is less consistent with the existence of two binding sites (Fig. 2f). As such, this acidic residue in the S3 segment has a stronger effect on ProTx-II binding when mutated in the chimaera versus Cav3.1. However, this can be explained by the presence of four potential binding sites within the Cav3.1/Kv2.1 chimaera, as opposed to only one in Cav3.1. Similar results have been observed for Nav/Kv chimaeras^36^. We also examined the acidic residue in domains I and II. The E198A domain I Cav3.1 mutant exhibits ProTx-II sensitivity similar to the wild type Cav3.1 channel (data not shown). We were not able to obtain functional channels with the domain II E855A Cav3.1 mutant. Overall, these results suggest that D1372 in domain III of Cav3.1 is an important residue for ProTx-II binding.

### PaTx-1, GsAF-I, and GsAF-II modulate Cav3.1

**Figure 3:**
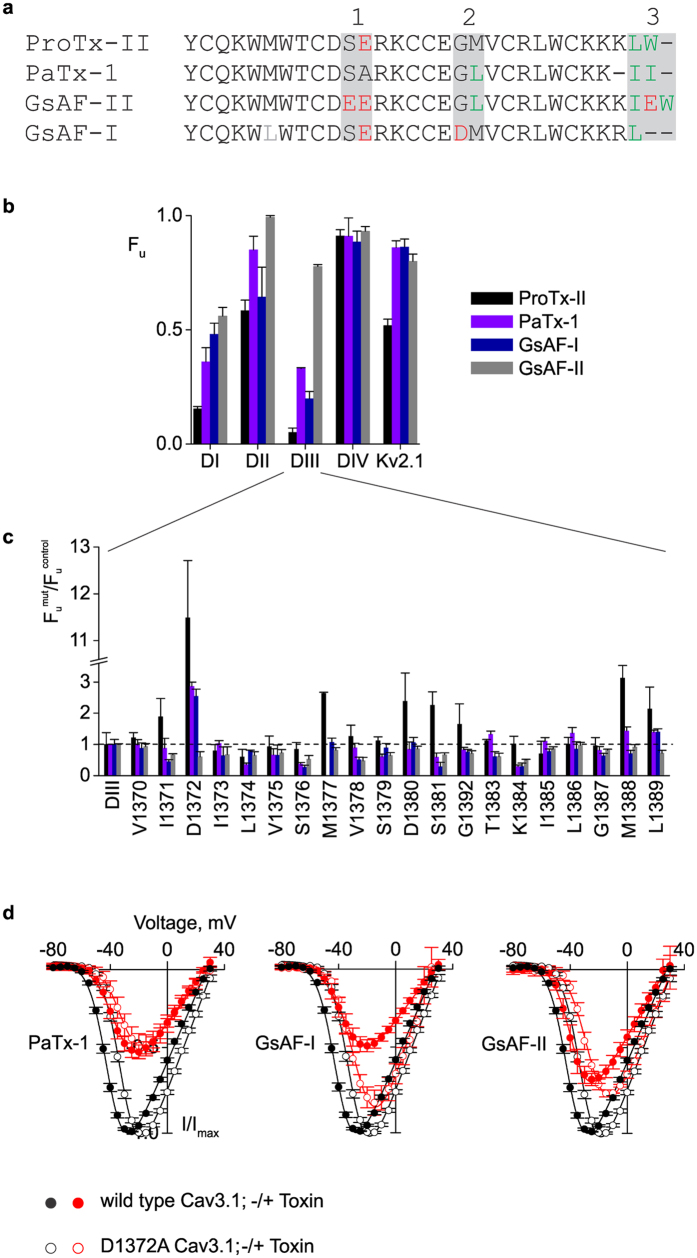
Tarantula toxins effects on Cav3.1 and Cav3.1/Kv2.1 chimaeras. (**a**) Sequence alignment of ProTx-II and three tarantula gating-modifier toxins. Regions of amino acid differences (sites 1–3) are highlighted in grey. Acidic residues are shown in red and hydrophobic residues in green. (**b**) Relative affinity of toxins for Kv2.1 and Cav3.1/Kv2.1 chimaeras. Data points are mean ± SEM (n = 6–9). (**c**) Alanine scan of DIII chimaera. Fraction of uninhibited current in the presence of ProTx-II, PaTx-1, GsAF-I, and GsAF-II for alanine mutants (F_u_^mut^) normalized to DIII construct (F_u_^control^). Data points are mean ± SEM (n = 3–6). (**d**) Voltage-activation data and Boltzmann fits for wild type (solid circles) and D1372A (open circles) Cav3.1 channels, in the absence (black, same data as in Fig. 2d) and presence (red) of PaTx-1, GsAF-I, and GsAF-II. V_1/2_ values are −35 ± 0.7 mV, −34 ± 0.6 mV, and −38 ± 0.5 mV for the wild type channel, and −31 ± 0.6 mV, −25 ± 0.8 mV, and −25 ± 0.8 mV for the D1372A channel. Toxin concentration was 1.33 μM. Data points are mean ± SEM (n = 6).

ProTx-II is a promiscuous toxin that was first reported to bind to Nav channels only^31^. In an effort to expand the limited pharmacological profile of Cav channels, we asked whether other toxins known to bind to Nav or Kv channels could also bind to Cav channels. Through a sequence search using ProTx-II as reference, we found three tarantula toxins, PaTx-1, GsAF-I, and GsAF-II, that share high sequence homology with ProTx-II (Fig. 3a). PaTx-1, isolated from *Phrixotrichus auratus*, is a potent inhibitor of Kv4.3 and Kv4.2^42^, while GsAF-I and GsAF-II, isolated from *Grammostola spatulata*, are described as “analgesic peptides” that target several Nav channel isoforms^43^,^44^. All four toxins share six cysteines that form the inhibitory cysteine knot (ICK) motif, and differ from ProTx-II by only one or two amino acids within three small regions, referred to as sites 1–3 (Fig. 3a). Although the NMR structures for GsAF-I and GsAF-II have not yet been solved, 3D modeling using PaTx-1 as the template suggests that they share similar structures^45^,^46^.

To study the effects of PaTx-1, GsAF-I, and GsAF-II on Cav channels and to identify the possible binding sites, we screened these toxins on Cav3.1, Cav/Kv chimaeras, and alanine mutant channels (Fig. 3 and Table 1). PaTx-1, similarly to ProTx-II, causes a positive shift (+6 mV) in the half-activation voltage of wild type Cav3.1 (Fig. 3d), inhibits domains I and III of Cav/Kv chimaeras, though with less potency than ProTx-II (Fig. 3b), and has a reduced effect on the D1372A domain III chimaera (Fig. 3c). When tested on the D1372A Cav3.1 mutant, however, no shift was observed for PaTx-1, despite a 50% decrease in peak current (Fig. 3d). For GsAF-I, we also found that the half-activation voltage of wild type Cav3.1 channel is shifted by +6 mV (Fig. 3d), that domains I and III Cav/Kv chimaeras are most inhibited (Fig. 3b), and again with less potency than ProTx-II, and that the D1372A mutation in the domain III chimaera weakens its inhibitory effect (Fig. 3c). In addition, the peak current of the D1372A Cav3.1 channel is only decreased by 15–20% in the presence of GsAF-I, though the half-activation voltage is shifted by +6 mV (Fig. 3d).

Finally, we found that GsAF-II displays noticeably different effects on the tested channels, when compared to the other toxins. When tested on the wild type Cav3.1 channel, GsAF-II shifts the half-activation voltage by only +3 mV and decreases the peak current by 25% (Fig. 3d). Although GsAF-II does show some inhibition of the domain I and III chimaeras, it is significantly less, particularly for domain III (15-fold), when compared to ProTx-II (Fig. 3b, GsAF-II vs ProTx-II). Moreover, the D1372A domain III chimaeric mutant does not decrease GsAF-II inhibition. In fact, a minor increase in inhibition (≈2-fold) is observed (Fig. 3c, F_u_^mut^/F_u_^control^ ≈0.5). The half-activation voltage of the D1372A Cav3.1 mutant is shifted by +6 mV, similar to ProTx-II and GsAF-I (Fig. 3d). Overall, we find that PaTx-1, GsAF-I, and GsAF-II modulate Cav3.1 channels, and that PaTx-1 and GsAF-I appear to behave most similarly to ProTx-II. Altogether, these experiments show that all these toxins bind to Cav3.1 channels, although they do not produce identical effects. None of them caused changes in the Cav3.1 channel steady-state inactivation or recovery from inactivation (data not shown).

### Membrane Partitioning

**Figure 4:**
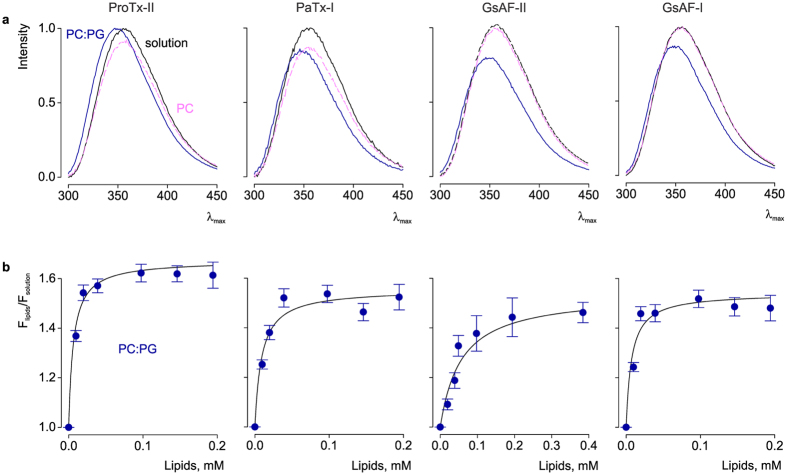
Interaction of tarantula toxins with lipid vesicles. (**a**) Intrinsic tryptophan fluorescence emission spectra of toxins in the absence (black) and presence of lipid vesicles composed of 1-palmitoyl-2-oleoyl-sn-glycero-3-phosphocholine (PC; pink) or a 1:1 ratio of 1-palmitoyl-2-oleoyl-sn-glycero-3-phosphocholine and 1-palmitoyl-2oleoyl-sn-glycero-3-[phosphor-rac-(1-glycerol) (PC:PG; blue). The lipid concentration was 1.5 mM. (**b**) Fluorescence intensity at 320 nm plotted vs. available lipid concentration (60% of total lipids). Curves are partition function fits with K_x_ = 10 ± 3 × 10^6^ and F_lipids_/F_solution_ = 1.65 ± 0.02 for ProTx-II; K_x_ = 6.1 ± 2 × 10^6^ and F_lipids_/F_solution_ = 1.32 ± 0.02 for PaTx-I; K_x_ = 9.8 ± 3 × 10^5^ and F_lipids_/F_solution_ = 1.53 ± 0.05 for GsAF-II, K_x_ = 7.6 ± 2 × 10^6^ and F_lipids_/F_solution_ = 1.27 ± 0.02 for GsAF-I. In all panels, data points are mean ± SEM (n = 3).

Previous studies have shown that several gating-modifier toxins act through a lipid bilayer pathway^22^,^23^,^25^,^26^,^33^,^47^. This mechanism requires an initial partitioning of the toxin in the membrane, prior to binding to the channel. Although depletion of ProTx-II from solution when incubated with lipid vesicles has been demonstrated^33^, the strength of this partitioning is unknown. Moreover, nothing is known about the potential partitioning of the three other toxins studied here. To address these questions, we used intrinsic tryptophan fluorescence (Figs 3a and 4) to follow the movement of toxins from an aqueous to a lipid environment^48^.

**Figure 5:**
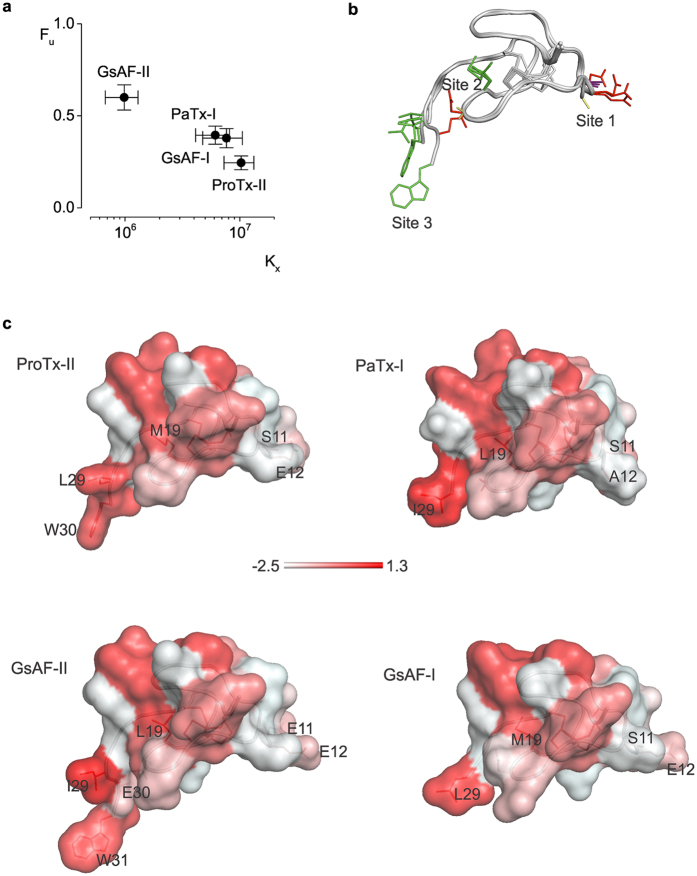
Comparison of related toxins on Cav3.1 inhibition and membrane partitioning strength. (**a**) Cav3.1 inhibition vs. strength of membrane partitioning. (**b**) ProTx-II, PaTx-1, GsAF-I, and GsAF-II structures superimposed based on their backbone fold. Side chains of residues within sites 1–3 are colored red (acidic), green (hydrophobic), purple (serine), and yellow (glycine and alanine). **(c)** Toxin surface profiles, colored based on the normalized Eisenberg hydrophobicity scale^64^. The most hydrophobic residues are in red, following a color gradient to the most hydrophilic residues in white.

In solution, all toxins display virtually identical tryptophan fluorescence emission spectra that peak at ≈355 nm. The spectra exhibit a shift (≈8 nm) to lower wavelengths (blue shift) upon incubation with saturating concentrations of a 1:1 mixture of neutral (PC) and anionic (PG) phospholipids (Fig. 4a). A blue shift is indicative of the toxin moving from an aqueous environment to a more restrictive lipid environment^48^. To quantify the strength of lipid partitioning, we calculated the mole fraction partition coefficient (K_x_) by measuring the changes in tryptophan fluorescence at 320 nm across a range of lipid concentrations (Fig. 4b)^22^,^48^. ProTx-II, PaTx-1, and GsAF-I have K_x_ values of 10 ± 3 × 10^6^, 6.1 ± 2 × 10^6^, 7.6 ± 2 × 10^6^, respectively, all of which indicate strong lipid partitioning^49^. In contrast, a twice-higher lipid concentration is needed to induce the same blue shift in the emission spectrum of GsAF-II (Fig. 4a,b). This corresponds to a K_x_ of 9.8 ± 3 × 10^5^, and therefore a 10-fold decrease in lipid partitioning strength compared to ProTx-II. Interestingly, GsAF-II inhibits Cav3.1 channels less than the other toxins, by a factor of two. This correlation between lipid partitioning strength and channel inhibition (Fig. 5a) has also been shown for gating-modifier toxins targeting Kv channels^22^. We also tested partitioning of these toxins in neutral lipids (PC) and observed no significant changes in tryptophan fluorescence (Fig. 4a). Despite high sequence homology among all four toxins, these data demonstrate that only a few changes in the amino acid sequence can significantly alter lipid partitioning properties, and thereby influence how toxins interact with the channel within the membrane.

## Discussion

In this study, we investigated the molecular interactions of tarantula toxins with the Cav3.1 channel and lipid vesicles. By constructing chimaeric Cav3.1/Kv2.1 channels, we showed that the four homologous S3–S4 segments of Cav3.1 can act as independent, functional motifs of voltage sensing and toxin binding. With these results, we now have a more complete picture of the conserved nature of the paddle motif in all three major voltage-gated ion channel types (Kv, Nav, and Cav). Furthermore, we showed that the differences in amino acid residues across the four Cav3.1 paddle sequences translate to distinct voltage-sensing properties, kinetics, and pharmacological profiles. Obviously, understanding how each paddle motif works in its own chimaera is not the same as understanding the function of four different and potentially interacting paddles within the wild type Cav3.1 channel. However, it is a necessary step towards identifying structural determinants of Cav3.1 gating and pharmacology. Using chimaeras is a straightforward and powerful approach for dissecting out the function of individual paddles and for identifying new pharmacological agents that could be used individually or in combination.

Few gating-modifier toxins have been identified for Cav3 channels. Moreover, the binding sites for these toxins have been unknown so far. Here, we focused on ProTx-II and found that it binds with highest affinity to domain I and III paddle chimaeras and that a 2:1 toxin-channel stoichiometry describes well the concentration-dependent inhibition of Cav3.1. ProTx-II was proposed to affect multiple steps along the activation pathway^29^, and this may be explained by the toxin binding to the voltage-sensors of domains I and III. Moreover, since no effect on Cav3.1 steady-state availability or recovery from inactivation was observed in the presence of ProTx-II^29^, we suggest that domains I and III do not contribute significantly to this channel property, but rather to channel activation. Our observations are consistent with what is known about Nav channels: domains I, II, and III are important for activation, whereas domain IV is involved in inactivation^50^,^51^,^52^. The picture derived so far from other studies in Cav channels is less complete, but suggests a similar division of labor between domains involved in activation vs. inactivation^3^. Interestingly, for the closely related Cav3.2 channel, a shift in the steady-state inactivation curve was observed in the presence of ProTx-II^30^, whereas no such effect was observed by us and others^29^ on Cav3.1 channels. This might seem puzzling, considering the high sequence homology between Cav3.1 and Cav3.2 with respect to the four S3–S4 sequences^53^. Nevertheless, these two channels display unique gating properties. For example, whereas the kinetics and voltage-dependency of activation and inactivation are very similar, the recovery from inactivation is more than 3-fold slower in Cav3.2^53^. Thus, it is possible that the increased time that Cav3.2 channels reside in an inactivated state facilitates a distinct, state-dependent binding of ProTx-II that explains the apparent differences in toxin effects between the two channel types. Similar chimaera and mutagenesis experiments between Cav3.2 and Kv2.1 would prove useful in comparing the structural correlates of inactivation between the two Cav3 isoforms.

To expand our understanding of the binding site of ProTx-II on Cav3.1, we alanine-scanned domain III, which was the most inhibited of all four chimaeras. For other toxin-channel pairs, multiple residues on the channel have been identified to be critically important for toxin binding, causing a strong, ≈10–25 fold decrease in inhibition when individually mutated to alanine^7^. This is not the case for the ProTx-II interaction with Cav3.1, where one single residue (D1372) causes an 8-fold decrease in inhibition upon mutation, with several additional residues making only small contributions. The analogous mutation in domain I (E198A) caused a similar effect, raising the possibility that acidic residues are important for ProTx-II binding^36^,^54^. Future studies could potentially derive more information from double or multiple mutants across the four Cav domains. Altogether, binding studies on Nav and Cav channels suggest that ProTx-II may require multiple, but individually weak interactions with the channel. A stronger dependence on lipid interactions for stabilizing the toxin-channel complex, and/or regions outside of the paddle contributing to toxin binding, could also explain these observations.

Since many gating-modifier toxins are promiscuous, we examined whether other known Nav or Kv channel toxins could inhibit Cav3 channels. A sequence homology search against ProTx-II yielded three tarantula toxins: PaTx-1, GsAF-I, and GsAF-II. Here, we show that these three previously reported gating-modifier toxins of Nav and Kv channels also target Cav3.1 channels and shift the half-activation voltage to more positive values. Although these toxins are almost identical in sequence, their effects are not the same. When comparing the effects of all four tarantula toxins, GsAF-II is an obvious outlier. The extent of Cav3.1 inhibition is roughly half of what is observed for the other toxins. Moreover, GsAF-II only very modestly inhibits the domain III paddle chimaera. Interestingly, when looking at a sequence alignment of all toxins against ProTx-II, it is clear that GsAF-II is the most different. Whereas ProTx-II, PaTx-1, and GsAF-I contain polar and hydrophobic residues at sites 1 and 3, respectively, GsAF-II has negatively charged glutamates at both sites (Figs 3a and 5b,c). These residues may cause weaker binding to Cav3.1, considering that an acidic residue on the channel (D1372) is important for binding ProTx-II-like toxins.

A recent structural and functional study that used chimaeras of ProTx-II and other related gating-modifiers also suggests that residues at sites 1 and 3 are particularly important for Nav channel interactions^45^. A chimaera made from the “tail” of ProTx-II and the “body” of VsTx-II was found to produce a 172-fold decrease in potency for Nav1.7 and a 285-fold decrease for Nav1.2. Interestingly, the body of VsTx-II is identical to that of GsAF-II, differing from the ProTx-II body at positions 11 and 19. The residue at position 19 was shown to be partially buried in a hydrophobic core in the NMR solution structure and is therefore unlikely to be directly involved in channel binding (Fig. 5b,c). This leaves the S to E variant at position 11 within site 1 as the most likely cause for the dramatic reduction in Nav1.7 and Nav1.2 binding. This is consistent with our predictions of E11 of GsAF-II contributing to the weaker inhibition of Cav3.1. Furthermore, a “PaTx1 body-ProTxII tail” chimaera was found to retain similar potency for Nav1.7 as ProTx-II, demonstrating the significant role of the C-terminus (site 3) in determining the potency for Nav1.7. Again, these experiments support our prediction that the differences between the tails of GsAF-II and ProTx-II contribute to a weaker Cav3.1 inhibition. Altogether, these experiments demonstrate that important information about the regions or residues critical for channel inhibition for a given toxin can be obtained from other toxins that differ in sequence by only a few amino acids. Although not as exhaustive as alanine-scanning mutagenesis of a given toxin, our approach can serve as an initial screening for identifying key interaction sites, without the challenges that accompany toxin mutagenesis.

Lastly, we investigated the interactions of the four tarantula toxins with lipid vesicles, and tested whether the few differences in amino acid sequence between these toxins could change the strength of lipid partitioning. We showed that three of these toxins, ProTx-II, PaTx-1, and GsAF-I, partition into lipid vesicles with equally high partitioning coefficients, while GsAF-II partitions with a 10-fold smaller K_x_. The cause for the poorer partitioning of GsAF-II may be the glutamate present within the tail domain. In contrast, only hydrophobic residues are found within the tail domain of the other three toxins. Considering that the tail domain of ProTx-II has been shown to be critical for potent inhibition of Nav channels^45^,^55^, it is possible that this region is significant for both channel binding and lipid partitioning. As discussed above, GsAF-II is not only the least potent inhibitor of Cav3.1, but also partitions the least, suggesting a correlation between lipid partitioning strength and Cav3.1 channel inhibition.

Overall, we have identified domains I and III in Cav3.1 channels as targets for ProTx-II, with the sites 1 and 3 in ProTx-II being the likely interaction sites. We have also identified three novel gating-modifier toxins for Cav3.1, expanding the pharmacological profile of these channels. Additionally, we have found that lipid partitioning strength is a potential predictor for how well a toxin inhibits Cav3.1 channels. Historically, understanding how toxins and channels interact has played a significant role in elucidating key biophysical and pharmacological properties of ion channels^52^,^56^,^57^,^58^,^59^. However, many questions remain unanswered. For example, why are certain toxins more promiscuous than others? What determines channel selectivity and potency? How do various channel and cellular components affect toxin action? Expanding the existing knowledge on toxin – channel interactions is crucial for answering these questions. In recent years, the key information brought by toxins has been cleverly exploited to engineer chimaeric and multimeric toxins to increase toxin potency or selectivity for a given channel^45^,^60^. This has clear implications on advancing the use of toxins as experimental tools and as therapeutic agents. Thus, investigating the complex effects that different toxins have on different channel types can provide valuable information to further expand both the traditional and novel uses of toxins.

## Methods

Toxins were purchased from Alomone Labs, Israel. All other chemicals were from Sigma-Aldrich, USA.

### Construction of Cav3.1/Kv2.1 chimaeras

Chimaeras and point mutations were made using sequential polymerase chain reaction (PCR) with Kv2.1 Δ7^34^,^61^ and rat α1G calcium channel Cav3.1 (XP_008766223.1). Cav3.1 D1372A was generated using site-directed mutagenesis (QuickChange II XL, Agilent, USA). The DNA sequence of all constructs and mutants was confirmed by DNA sequencing. Complementary RNA (cRNA) was synthesized using T7 polymerase (mMessage mMachine kit, Ambion, USA) after linearizing the DNA with appropriate restriction enzymes.

### Electrophysiology

All channel constructs were expressed in *Xenopus laevis* oocytes and studied 1–5 days after cRNA injection and incubation at 17 °C in (mM): 96 NaCl, 2 KCl, 5 HEPES, 1 MgCl_2_, 1.8 CaCl_2_, and 50 μg/ml gentamicin, pH 7.6 with NaOH, using the two-electrode voltage-clamp recording technique (OC-725C amplifier, Warner Instruments, USA) within a 150 μl recording chamber. Data were filtered at 5 kHz and digitized at 10 kHz using pClamp 10 software (Molecular Devices, USA). Microelectrode resistance was 0.3–1 MΩ when filled with 3 M KCl. For recording potassium currents, the external recording solution contained (in mM): 20 KCl, 80 NaCl, 10 HEPES, 1 MgCl_2_, and 0.3 CaCl_2_, pH 7.6 adjusted with NaOH. For recording Cav3.1 channel currents, the external recording solution contained (in mM): 5 Ba(OH)_2_, 100 NaCH_3_SO_3_, 10 HEPES, pH 7.6 adjusted with HCl. All experiments were performed at room temperature (~22 °C). Currents were corrected for leak and endogenous currents by subtracting the calculated linear leak at negative potentials, and by subtracting the residual current measured after blocking the Kv channels with 1 μM agitoxin-2, and Cav channels with 1 mM CdCl_2_.

Voltage–activation data were obtained by measuring tail currents for Kv channels or steady-state currents for Cav channels. For Kv experiments, the data were fitted using the equation I/I_max_ = [1 + exp(−zF(V − V_1/2_)/RT)]^−1^, where I/I_max_ is the normalized tail current amplitude, z is electrical charge, V_1/2_ is the half-activation voltage, F is Faraday’s constant, R is the gas constant, and T is the absolute temperature. For Cav experiments, the data were fitted using the equation I/I_max_ = ((V − V_rev_)G_max_)/(1 + exp(−zF(V − V_1/2_)/RT)), where I/I_max_ is the normalized peak current amplitude, V_rev_ is the current reversal voltage, G_max_ is the maximum conductance, and V_1/2_ is the half-activation voltage.

### Estimating toxin occupancy of channels

The occupancy of closed or resting channels by toxins was examined at negative holding voltages where the open probability is low. The fraction of unbound channels (F_u_) was estimated using depolarizations that are too weak to fully open toxin-bound channels, as previously described^7^,^8^,^9^,^10^,^19^,^34^,^62^,^63^. We recorded voltage-activation relationships in the absence and presence of different concentrations of toxins. We calculated the ratio of currents (I/I_0_) recorded in the presence (I) and absence (I_0_) of toxin for various depolarizing voltages. F_u_ was obtained as the value of I/I_0_ measured in the plateau phase at voltages where toxin-bound channels do not open (Fig. 2e). For all the experiments, voltage protocols were adjusted to have a well-defined plateau phase in the I/I_0_-voltage relationship. The apparent K_d_ for toxin inhibition was calculated assuming *n* equal and independent toxin-binding sites per channel, with single occupancy (n = 1) being sufficient to inhibit opening in response to weak depolarizations: K_d_ = ((1/(1 − F_u_^1/n^)) −1) × [Toxin]. For all potassium conducting channels (Kv2.1 and chimaeras), n was constrained to 4 (Table 1). For Cav channels, n and K_d_ were fitted together as free parameters.

### Toxin partitioning in lipid vesicles

Large unilamellar vesicles (LUVs) were prepared from a mix of 1:1 molar ratios of PC (1-palmitoyl-2oleoyl-sn-glycero-3-phosphocholine) and PG (1-palmitoyl-2oleoyl-sn-glycero-3-[phosphor-rac-(1-glycerol)]) (Avanti Polar Lipids, USA), dried from a chloroform solution under nitrogen stream. The lipid film was rehydrated in a buffer containing (in mM): 10 HEPES, 1 EDTA, pH 7.6 adjusted with NaOH, and then extruded through polycarbonate filters with a 100 nm pore size (Millipore, Bedford, MA, USA). LUVs were added to a solution of toxin (2 μM final concentration), maintained at 25 °C with continuous stirring in a quartz cuvette with 1 cm path length (2 ml total volume). Fluorescence spectra (averaging two spectra) were recorded between 300 and 400 nm (5 nm band pass, 0° polarizer) using an excitation wavelength of 280 nm (5 nm band pass, 90° polarizer) (SPEX FluoroMax 3 spectrofluorometer, HORIBA) and corrected for vesicle scattering. For calculating mole-fraction partitioning coefficients (K_x_), fluorescence intensity (F) at 320 nm was measured and normalized to the zero-lipid fluorescence intensity (F_0_)^22^,^48^. K_x_ was calculated using the equation: F⁄ F_0_(L) = 1 + (F⁄ F_0_^max^ − 1)K_x_[L]/([W] + K_x_[L]), where F⁄ F_0_(L) is the change in fluorescence intensity for a given concentration of lipid, F⁄(F_0_^max^) is the maximum fluorescence increase at high lipid concentrations, [L] is the average available lipid concentration (60% of total lipid concentration), and [W] is the molar concentration of water (55.3 M).

### Molecular Modeling

Molecular models of ProTx-II, GsAF-I, and GsAF-II were created based on the NMR structure of PaTx-1 (PDB 1v7f) by aligning and minimizing RMSD between the cysteine α-carbons of the toxins (PyMOL, DeLano Scientific).

## Additional Information

**How to cite this article**: Salari, A. *et al.* Molecular Interactions between Tarantula Toxins and Low-Voltage-Activated Calcium Channels. *Sci. Rep.* **6**, 23894; doi: 10.1038/srep23894 (2016).

## Figures and Tables

**Table 1:** Gating properties and toxin inhibition of Kv2.1 and Cav3.1/Kv2.1 chimaeric constructs.

Construct	V_1/2_ (mV)	z	K_d_ (μM)			
			ProTx-II	PaTx-1	GsAF-I	GsAF-II
Kv2.1	−5.8 ± 1	2.6 ± 0.3	7.4 ± 0.2	17 ± 0.3	36 ± 0.7	23 ± 0.5
DI	4.4 ± 0.9	3.06 ± 0.4	2.2 ± 0.07	2.3 ± 0.2	6.6 ± 0.3	8.5 ± 0.3
E198A	23 ± 2	2.8 ± 0.1	7.7 ± 0.2	5.4 ± 0.2	19 ± 0.6	>50
DII	25 ± 0.7	2.9 ± 0.1	9.2 ± 0.3	16 ± 0.5	11 ± 1	>50
E855A	8 ± 0.5	2.4 ± 0.1	30 ± 0.7	>50	>50	>50
DIII	−1.3 ± 1	2.6 ± 0.1	1.2 ± 0.2	2.1 ± 0.01	2.7 ± 0.2	20 ± 0.2
V1370	−4 ± 1	1.7 ± 0.1	1.3 ± 0.09	2 ± 0.1	2.4 ± 0.2	16 ± 0.6
I1371	21 ± 1	1.3 ± 0.1	1.6 ± 0.2	1.8 ± 0.3	1.6 ± 0.1	7.6 ± 0.1
D1372	11 ± 1	2.1 ± 0.2	9.1 ± 0.4	47 ± 1.1	7.2 ± 0.3	6.4 ± 0.7
I1373	−12 ± 1	2.2 ± 0.1	1.07 ± 0.1	2.1 ± 0.1	2 ± 0.4	7.7 ± 1
L1374	−9 ± 1	1.7 ± 0.1	0.9 ± 0.2	0.9 ± 0.07	2.3 ± 0.03	7.2 ± 0.5
V1375	−14 ± 1	1.7 ± 0.1	1.1 ± 0.1	1.4 ± 0.2	2 ± 0.5	8.8 ± 0.5
S1376	13 ± 1	1.1 ± 0.1	1.1 ± 0.1	0.9 ± 0.06	1.2 ± 0.1	5.4 ± 0.5
M1377	28 ± 1	1.2 ± 0.1	2 ± 0.1	ND	2.8 ± 0.2	11 ± 0.4
V1378	8 ± 2	1.1 ± 0.1	1.3 ± 0.2	1.8 ± 0.1	1.7 ± 0.1	4.7 ± 0.4
S1379	−4 ± 1	1.1 ± 0.1	1.2 ± 0.7	1.3 ± 0.1	2.4 ± 0.2	7.1 ± 0.4
D1380	13 ± 2	1.0 ± 0.1	1.9 ± 0.3	1.8 ± 0.2	2.8 ± 0.2	13 ± 0.5
S1381	25 ± 1	1.1 ± 0.1	1.8 ± 0.1	1.3 ± 0.1	1.3 ± 0.3	7.6 ± 0.2
G1392	26 ± 1	1.5 ± 0.1	1.5 ± 0.3	1.7 ± 0.08	2.2 ± 0.1	8.4 ± 0.4
T1383	7 ± 1	1.3 ± 0.1	1.2 ± 0.02	2.9 ± 0.1	1.9 ± 0.2	6.5 ± 0.4
K1384	5 ± 2	0.9 ± 0.05	1.2 ± 0.1	0.8 ± 0.07	1.3 ± .02	5 ± 0.07
I1385	−7 ± 2	1.1 ± 0.1	1 ± 0.2	2.3 ± 0.1	2.2 ± 0.1	13 ± 0.3
L1386	4 ± 2	0.8 ± 0.05	1.2 ± 0.1	3 ± 0.2	2.4 ± 0.3	19 ± 0.7
G1387	−9 ± 1	1.9 ± 0.1	1.1 ± 0.1	1.7 ± 0.1	2 ± 0.1	11 ± 0.1
M1388	−4 ± 1	1.3 ± 0.1	2.3 ± 0.1	3.2 ± 0.1	2.1 ± 0.1	15 ± 0.5
L1389	−28 ± 2	1.4 ± 0.1	1.8 ± 0.3	3.1 ± 0.07	3.5 ± 0.1	8.6 ± 0.4
DIV	−87 ± 1	3.4 ± 0.3	>50	28 ± 1.2	>50	>50
